# Supplementary material for: Biomarkers for Non-Invasive Stratification of Coronary Artery Disease and Prognostic Impact on Long-Term Survival in Patients with Stable Coronary Heart Disease
Source: Nutrients. 2022 Aug 20;14(16):3433. doi: 10.3390/nu14163433 (PMC9413764; doi:10.3390/nu14163433)
Supplement: Supplementary file 1 [file nutrients-14-03433-s001.zip › nutrients-1794583-supplementary.pdf]

Study population *N* **= 3.305** patients (**males** *N* **= 2.122** and **females** *N* **= 1.183**) admitted for clinically suspected chronic coronary syndrome, i.e., CAD and undergoing first-time coronary angiography.

↓

Exclusion of **233** patients (of which 121 were excluded for unknown angiographic findings and 112 for hs-cTnT ≥ 52 pg/ml, indicating acute coronary syndrome).

↓

N= **3.072** patients (**males** *N* **= 1.966**, 64%; and **females** *N* **= 1.106**, 36%) were used for the analysis. The median follow up-time for survival analysis was 10 years (range 0-14).

↓

Groups studied:

CAD0 (no coronary sclerosis),
CAD1 (non-obstructive coronary sclerosis, i.e., plaques with < 50% luminal reduction),
obstructive CAD2 (≥ one stenosis ≥ 50% luminal reduction).

We primarily compared:
CAD0 + 1 vs. CAD2 (GC1: group comparison 1),
CAD0 vs. CAD1 + 2 (GC2: group comparison 2).

Figure S1. Flow chart of the study design

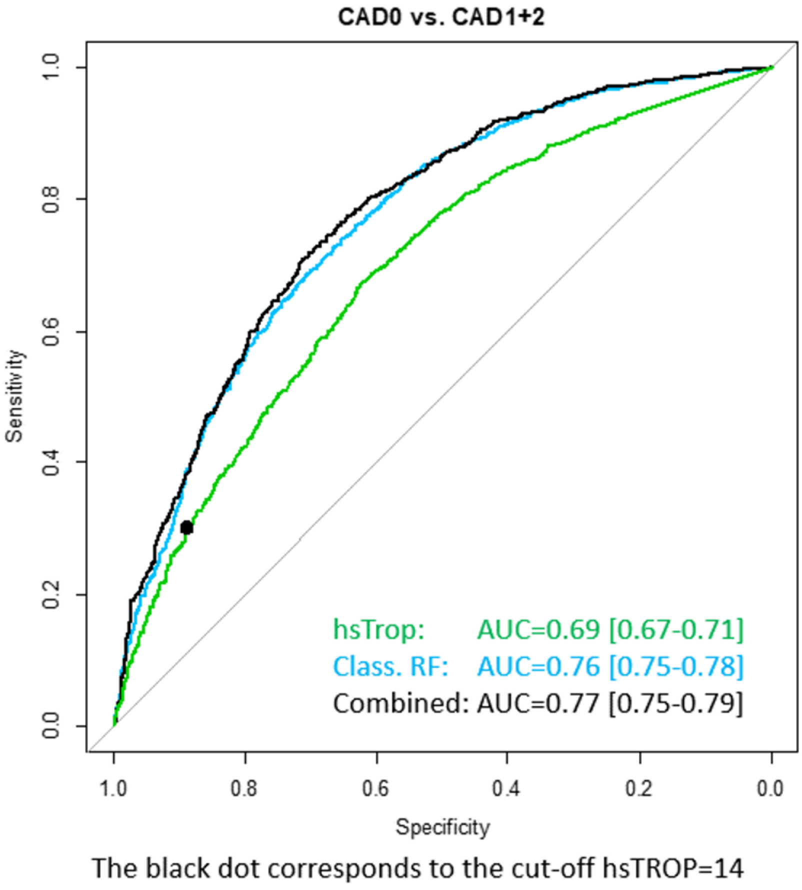

Figure S2. ROC analysis of the prediction of significant CAD (CAD0 vs. CAD1 + 2 (CG2) group comparison 2). Hs-cTnT alone (green), classical risk factors (blue), and classical risk factors + hs-cTnT (black). Hs-cTnT levels alone has an AUC of 0.69 (95% CI: 0.67–0.71). The classical risk factors—age, sex, BMI, smoking, diabetes, LDL-C, HDL-C, hypertension, WHR, triglycerides, and eGFR without hs-cTnT—showed an AUC of 0.76 (0.75–078) and 0.76 (0.74–0.78), respectively. The addition of hsTNT to the classical risk factors revealed a significant increment in the AUC to 0.77 (95% CI: 0.75–0.79, $p = 0.0013$) and 0.77 (95% CI: 0.75–0.78, $p = 0.029$), respectively. GC: group comparison, AUC: area under the curve, CI: confidence interval.
